# Clinical effectiveness of a brief educational intervention in Type 1 diabetes: results from the BITES (Brief Intervention in Type 1 diabetes, Education for Self-efficacy) trial

**DOI:** 10.1111/j.1464-5491.2008.02607.x

**Published:** 2008-12

**Authors:** J T George, A P Valdovinos, I Russell, P Dromgoole, S Lomax, D J Torgerson, T Wells, J C Thow

**Affiliations:** York Hospital and Hull-York Medical School (HYMS)York, UK; * División de Innovación en Servicios de Salud, Coordinación de Políticas de Salud, Dirección de Prestaciones Médicas, Instituto Mexicano del Seguro SocialMexico; † University of WalesBangor; ‡ Independent Nurse PractitionerYork; § York Trials Unit, University of YorkYork; ¶ Department of Psychology, City HospitalsSunderland, UK

**Keywords:** diabetes treatment satisfaction, educational intervention, glycaemic control, self-efficacy, Type 1 diabetes

## Abstract

**Aims:**

Intensive 5-day educational interventions for people with Type 1 diabetes have shown improved outcomes in a number of European studies. The aim was to assess the effectiveness of a brief (2.5 days) psycho-educational intervention.

**Methods:**

Our randomized trial in a secondary-care setting had 54 and 60 participants allocated to intervention and control groups, respectively. Primary outcomes were HbA_1c_ and severe hypoglycaemia. Secondary outcomes were blood pressure, weight, height, lipids and psychometric profile.

**Results:**

HbA_1c_ showed no statistically significant change at 3 months [difference = 0.01, 95% confidence interval (CI) –0.23, 0.26, *P* = 0.92], 6 months (difference = –0.06, 95% CI –0.32, 0.20, *P* = 0.67) and 12 months (difference = 0.01, 95% CI –0.30, 0.32, *P* = 0.94). Incidence of severe hypoglycaemia (per patient per year) in the intervention group (0.41) and control group (0.48) was not statistically different. Treatment satisfaction improved at 3 months (difference = 9.4, 95% CI 5.2, 13.6, *P* = 0.0005), 6 months (difference = 10.4, 95% CI 6.0, 14.8, *P* = 0.0005) and 12 months (difference = 7.1, 95% CI 2.1, 12.1, *P* = 0.006). The ‘Managing psychological aspects’ and ‘Setting and achieving goals’ dimensions of the Diabetes Empowerment Scale also showed significant improvement at 3, 6 and 12 months. Diabetes Knowledge Test, Illness Perception Questionnaire, Hypoglycaemia Fear Scale and Short Form 36 showed no significant change.

**Conclusions:**

This brief intervention had no significant impact on HbA_1c_ or severe hypoglycaemia, but improved diabetes treatment satisfaction and patient empowerment. Current Controlled Trials ISRCTN75807800.

## Introduction

Self-management is a critical component of preventive care in people with diabetes [1,2]. Intensive educational interventions providing self-management skills for people with diabetes have reduced blood glucose concentration in a number of studies [3–8]. This, in turn, reduces rates of complications [9,10].

In the UK, the Dose Adjustment for Normal Eating (DAFNE) group was the first to deliver an effective educational programme for Type 1 diabetes [8]. DAFNE is based on a successful in-patient programme developed in Düsseldorf, Germany [3] and is delivered in 35 h over five consecutive days. Many diabetes teams in the UK have since developed relatively brief educational programmes delivered in 15–24 h over 4–6 weeks. [11] The effectiveness of these interventions has not been assessed in randomized controlled trials.

We report a pragmatic randomized controlled trial carried out in a realistic clinical setting to determine the effectiveness of a brief (2.5 days) psycho-educational intervention for self-management in people with Type 1 diabetes.

## Methods

### Intervention

#### Philosophy and principles

BITES intervention is based on psychological theories of self-regulation [12,13] and social learning [14,15]. The underlying philosophy of the programme is that of patient empowerment [16,17] and promoting self-management of diabetes. In the delivery of BITES, we used basic principles of adult learning: conceptualization (in the classroom), observation (classroom and home), experimentation leading to expertise (home), and reflection (classroom and at home after the intervention).

BITES also included use of cognitive behavioural techniques. We sought to give participants insight into motivational principles they could apply. Rationale was provided for using insulin that is consistent with their illness cognitions (i.e. their view of their diabetes) and enhancement of their self-efficacy (i.e. their feeling of control over their diabetes). Changing negative to positive thoughts and maintaining changes were discussed. BITES also explored coping, control and strategies for prioritizing diabetes.

#### Design

A multiprofessional team comprising a consultant diabetologist, diabetes specialist nurse, specialist diabetes dietician and clinical health psychologist designed the BITES intervention.

#### Educational goals and learning outcomes

The BITES curriculum covers three educational themes: understanding carbohydrates and diet, understanding insulin adjustment and giving patients the skills and confidence to self-manage diabetes. Learning outcomes covered under these themes are listed in Table 1.

**Table 1:** Educational goals and learning outcomes of the BITES intervention

**1)Understanding carbohydrates and diet**
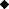 Analysing food content
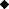 Dietary liberalization
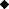 Understanding glycaemic index
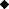 Practical carbohydrate counting
**2)Understanding insulin adjustment**
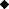 Insulin and its effects
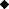 Role of pre- and post-meal blood glucose testing
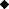 Insulin:carbohydrate ratios
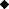 Impact of stress, exercise, and environment on blood glucose and insulin requirement
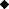 Adjusting insulin for carbohydrates without using algorithm
**3)Giving patients the confidence to self-manage diabetes**
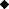 Avoiding hypos
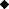 Sick-day rules
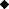 Complications
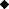 Skills to effect and maintain change.

Pre- and post-meal blood glucose monitoring were promoted with participants encouraged to self-adjust insulin doses according to their carbohydrate intake, level of activity, anticipation of exercise, climatic change on holiday, time zones and the type of insulin regime. Participants were provided with a ready-reckoner table to help estimate their initial carbohydrate:insulin ratios based on their total daily insulin requirement. Further refinement of carbohydrate:insulin ratios were made on an individual basis based on pre- and post-meal testing. Participants were not given fixed algorithms to titrate insulin doses. Carbohydrate estimation was taught early in the course, with insulin titration skills provided in a subsequent session, thus ensuring sufficient time to practise carbohydrate skills, before introducing titration of insulin. The timetable of the course listing its contents is provided in Table 2.

**Table 2:** Syllabus and timetable for brief educational intervention, BITES

Day 1 in week 1 (09.00–16.00 h)	Day 2 in week 2 (09.00–16.00 h)	Day 3 in week 6 (09.00–13.00 h)
1. Introduction	6. Feedback from last session & Workbook	11. Feedback from last session & Workbook
2. Freedom and control in day-to-day diabetes	7. Using insulin as a tool	12. Fine-tuning—activity, eating out & illness
3. Principles of healthy eating	8. Understanding insulin adjustment	13. ‘Going for Gold’ (motivational video)
4. Analysing food content & estimating carbohydrates	9. Blood glucose monitoring as a tool (including testing before & after meals)	14. Maintaining change
5. Group exercise—analysing food content	10. Exercise in ‘free diet’—combined food analysis and insulin adjustment	
**Workbook**	**Workbook**	
Food analysis—practical exercises & daily comments	Food analysis & insulin adjustment— daily comments	
1 scheduled phone support call	1 scheduled phone support call	

#### Delivery

A specifically trained diabetes specialist nurse and a specialist diabetes dietician facilitated delivery in six groups of 8–10 participants as a 2.5-day course over a 6-week period using pre-approved educational material. Gaps between sessions were intended to help participants consolidate their understanding, allowing time to practise, reflect and develop skills on an individual basis.

#### Learning methods

Sessions were interactive and reflection in between sessions encouraged. Group-based problem solving exercises were used during the sessions. Participants completed a workbook in between sessions and received feedback from peers and healthcare professionals at the following session.

Participants were also introduced to a fictitious individual with diabetes whom they mentored throughout the course and discussed helping them with change. This allowed the participants to offer advice on the need for change without excessive personalization, and provided continuity as new concepts and learning were reviewed.

#### Assurance of treatment fidelity

Treatment fidelity was ensured by the use of a written curriculum, pre-approved educational materials, observation by an independent researcher and using the same set of health professionals to deliver the intervention.

### Participants and protocol

Participants were recruited from people with diabetes attending our specialist diabetes service in a hospital setting. Eligibility criteria were: Type 1 diabetes for > 12 months, multiple injection therapy for ≥ 2 months, minimum age of 18 years and ability to read and write. York Research Ethics Committee (Ref: 01/08/016) approved the study protocol.

Postal invitations were sent to eligible participants along with information about the study. Willing respondents were seen at randomization clinics, where the research team verified eligibility and obtained written informed consent. An independent evaluator then allocated participants using block randomization (block size = 6) to intervention or control groups using sealed envelopes in strict ascendant order. The intervention group was offered the course in six groups of 8–10. Participants in both groups attended the four assessments at baseline, 3, 6 and 12 months, plus their usual care. After the course, usual care continued.

The control group were seen in their usual diabetes clinic, in addition to their study appointments. Patients had access to Diabetes Specialist Nurses and Specialist Diabetes Dietician, and access to the Clinical Health Psychologist by referral. The control group received the full course 12 months later.

The full protocol for the study is available as an open source publication [18].

### Primary outcomes

Haemoglobin A_1c_ (HbA_1c_) was measured (Diabetes Complications Control Trial aligned) and severe hypoglycaemia recorded at baseline, 3, 6 and 12 months. Severe hypoglycaemia was defined as a recorded episode in which the patient required assistance with treatment and either documented blood glucose < 2.7 mmol/l or detected clinical signs that required oral carbohydrate administered by a third party, subcutaneous glucagon or intravenous glucose.

### Secondary outcomes

Blood pressure, weight, height, body mass index (BMI), lipid profile and the number of daily insulin injections were used as secondary end-points. Participants were asked to complete a psychosocial and knowledge questionnaire (221 items) with Cronbach α 0.60–0.94. The questionnaire included the following scales: Medical Outcomes Study 36-Item Short-form Health Survey (SF-36) to measure general health status [19]; Illness Perception Questionnaire (IPQ) to measure participants’ perceptions of their diabetes [20]; Diabetes Knowledge Test (DKT) to assess participants’ knowledge about diabetes [21]; Diabetes Empowerment Scale (DES) to measure participants’ self-efficacy in caring for their diabetes [17]; Diabetes Treatment Satisfaction Questionnaire (DTS-Q) to measure participants’ satisfaction with their current treatment for diabetes [22]; Hypoglycaemia Fear Scale (HFS) to measure participants’ worries about hypoglycaemia and their associated behaviours [23]; and Diabetes Health Profile (DHP) with its three subscales: psychological distress, barriers to activity, and disinhibited eating [24].

### Statistical analysis

We used intention to treat analysis of covariance (ancova) adjusting for baseline scores. To have 80% power to detect a 1.0% difference in HbA_1c_, which we deemed to be of clinical significance, required us to recruit at least 90 participants. A *P*-value of < 0.05 was considered to be statistically significant.

### Technical data

HbA_1c_ was analysed by an ion-exchange high-performance liquid chromatography method [25] (Tosoh Medics, Foster City, CA, USA). The normal range for healthy subjects (mean ± 2 sd) is 4.4–6.1%. Blood pressure, weight and height (and calculated BMI in kg/m^2^) were measured by standardized methods.

## Results

**Figure 1:**
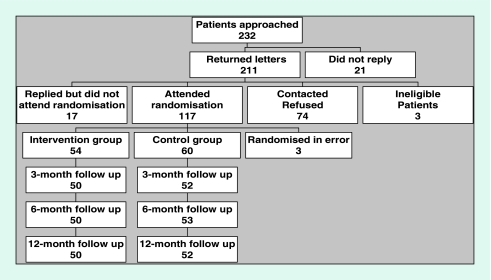
Participant recruitment and flow in the BITES study.

Patients meeting eligibility criteria (*n* = 232) were invited by post to participate. Of the 117 patients attending randomization, 54 were allocated to the intervention group and 60 to the control group. Figure 1shows the flow of participants in the study.

Analysis was by intention to treat. Characteristics of participants in the two groups were comparable at entry (Table 3). No adverse events were reported.

**Table 3:** Comparability of intervention, control and refusal groups at baseline

Variable	Intervention group (*n* = 54)	Control group (*n* = 60)	Refusals (*N* = 74)
Male (%)	50	40	50
Age (years)	41 ± 10	41 ± 12	41 ± 14
Duration of diabetes (years)	19.7 ± 12.7	19.4 ± 11.0	19.0 ± 11
HbA_1c_ (%)	8.7 ± 1.51	8.7 ± 1.13	8.8 ± 1.6
BMI (kg/m^2^)	26.3 ± 3.74	27 ± 4.17	26.6 ± 4.0
Total cholesterol (mmol/l)	5.0 ± 1.05	5.2 ± 0.83	NA
Triglycerides (mmol/l)	1.7 ± 1.30	1.9 ± 1.43	NA
HDL-cholesterol (mmol/l)	1.8 ± 0.50	1.8 ± 0.83	NA
LDL-cholesterol (mmol/l)	2.4 ± 0.85	2.6 ± 0.80	NA

Values are expressed as means (sd).

BMI, body mass index; HDL, high-density lipoprotein; LDL, low-density lipoprotein; NA, not available.

### Primary outcomes

Although HbA_1c_ fell at 3 months in both groups from 8.7 to 8.4%, there were no statistically significant differences between the groups at 3 months [difference = 0.01, 95% confidence interval (CI) of difference –0.23, 0.26, *P* = 0.92], 6 months (difference = –0.06, 95% CI –0.32, 0.20, *P* = 0.67) or 12 months (difference = 0.01, 95% CI –0.30, 0.32, *P* = 0.94). There were 0.41 episodes of severe hypoglycaemia (per patient per year) in the intervention group and 0.48 episodes in the control group with no statistical difference at 12 months. Seven participants in the intervention group and six in the control group had a baseline HbA_1c_ < 7.5%. An analysis was performed excluding these participants and no statistically significant differences were observed. A summary of biophysical outcomes is provided in Table 4.

**Table 4:** Difference between means of bio-physical outcomes obtained between intervention and control groups at 3, 6 and 12 months

	3 months		6 months		12 months	
Outcomes	Difference between means (95% CI)	*P*	Difference between means (95% CI)	*P*	Difference between means (95% CI)	*P*
HbA_1c_ (%)	0.01 (–0.23, 0.26)	0.92	–0.06 (–0.32, 0.20)	0.67	0.01 (–0.30, 0.32)	0.94
Cholesterol (mmol/l)	N/A	N/A	–0.12 (–0.34, 0.10)	0.28	–0.04 (–0.18, 0.26)	0.70
Triglycerides (mmol/l)	N/A	N/A	–0.28 (–0.64, 0.08)	0.13	–0.13 (–0.42, 0.16)	0.37
Severe hypoglycaemia*	N/A	N/A	N/A	N/A	–0.05 (–0.61, 0.50)	0.85
Body mass index (kg/m^2^)	–0.01 (–0.35, 0.33)	0.96	–0.07 (–0.48, 0.34)	0.74	0.07 (–0.56, 0.42)	0.77
Total daily insulin dose (units)	0.33 (–2.17, 2.84)	0.79	2.33 (–0.46, 5.11)	0.10	0.29 (–3.30, 3.88)	0.87

Fifty-four patients in intervention group and 60 patients in the control group.

* Severe episodes per annum.

### Secondary outcomes

Lipids, blood pressure, use of insulin and BMI demonstrated no statistical significance between the two groups (Table 3). In both groups, 94% of participants were injecting insulin four or five times per day at baseline and 98% at 12 months.

DKT, IPQ, HFS and SF-36 scores did not show any statistically significant improvement. In DH, no statistically significant findings were seen between the two groups except in the ‘barriers to activity’ dimension, which improved at 3 months (difference = –4.5, 95% CI –7.4, –1.5, *P* = 0.003), 6 months (difference = –4.6, 95% CI –8.4, –0.9, *P* = 0.02) and 12 months (difference = –3.5, 95% CI –6.4, –0.7, *P* = 0.02). The ‘Managing psychological aspects’ dimension of DES showed improvement in the intervention group at 3 months (difference = 3.7, 95% CI 1.2, 6.1, *P* = 0.004), 6 months (difference = 3.9, 95% CI 1.3, 6.4, *P* = 0.003) and 12 months (difference = 4.6, 95% CI 2.1, 7.2, *P* = 0.0005). The ‘Setting and achieving goals’ dimension of DES also showed a positive change at 3 months (difference = 3.1, 95% CI 0.6, 5.6, *P* = 0.02), 6 months (difference = 3.8, 95% CI 0.9, 6.7, *P* = 0.01) and 12 months (difference = 3.7, 95% CI 0.6, 6.9, *P* = 0.02).

Treatment satisfaction on DTS-Q improved at 3 months (difference = 9.4, 95% CI 5.2, 13.6, *P* = 0.0005), 6 months (difference = 10.4, 95% CI 6.0, 14.8, *P* = 0.0005) and 12 months (difference = 7.1, 95% CI 2.1, 12.1, *P* = 0.006). Psychological outcomes are summarized in Table 5.

**Table 5:** Psychosocial outcomes at 3, 6 and 12 months*

		3 months		6 months		12 months	
Outcomes	Dimensions	Difference between means (95% CI)	*P*	Difference between means (95% CI)	*P*	Difference between means (95% CI)	*P*
Short Form 36	Physical health (PCS)	1.4 (–1.6, 4.3)	0.35	2.2 (–0.7, 5.0)	0.14	1.9 (–0.8, 4.6)	0.17
	Mental health (MCS)	1.1 (–2.6, 4.8)	0.57	0.78 (–2.6, 4.2)	0.65	0.33 (–3.3, 4)	0.86
Diabetes Empowerment Scale	Managing psychological aspects	3.7 (1.2, 6.1)	0.004	3.9 (1.3, 6.4)	0.003	4.6 (2.1, 7.2)	0.0005
	Setting and achieving goals	3.1 (0.6, 5.6)	0.02	3.8 (0.9, 6.7)	0.01	3.7 (0.6, 6.9)	0.02
	Dissatisfaction and readiness to change	2.1 (–1.0, 5.1)	0.19	0.20 (–2.7, 3.1)	0.89	2.87 (–0.3, 6.1)	0.08
Hypoglycaemia Fear Scale	Behaviour	–0.29 (–3.4, 2.8)	0.85	−.01 (–2.9, 2.9)	0.99	–1.2 (–4.2, 1.9)	0.45
	Worry	–3.0 (–7.0, 1.1)	0.15	–2.4 (–7.2, 2.4)	0.33	–1.4 (–6.2, 3.4)	0.57
Diabetes Treatment Satisfaction	Treatment satisfaction	9.4 (5.2, 13.6)	0.0005	10.4 (6.0, 14.8)	0.0005	7.1 (2.1, 12.1)	0.006
	Hyperglycaemia	–4.1 (–12.0, 3.8)	0.31	–3.2 (–10.8, 4.4)	0.40	2.0 (–5.3, 9.4)	0.59
	Hypoglycaemia	0.9 (–7.0, 8.9)	0.82	–0.2 (–7.7, 7.3)	0.95	3.5 (–3.7, 10.8)	0.34
Illness (Diabetes) Perception Questionnaire	Timeline	0.5 (–2.97, 3.98)	0.77	2.6 (–0.72, 5.9)	0.12	–3.2 (–7.2, 0.8)	0.11
	Consequences	1.5 (–1.75, 4.81)	0.36	–3.6 (–8.0, 0.8)	0.11	–4.1 (–8.7, 0.5)	0.08
	Cure/control	1.9 (–1.02, 4.80)	0.20	3.0 (–0.12, 6.1)	0.06	3.7 (–0.3, 7.8)	0.07
	Causes	–0.58 (–3.6, 2.48)	0.71	2.6 (–1.3, 6.6)	0.18	0.64 (–2.7, 4)	0.70
Diabetes Health Profile	Psychological distress	–2.0 (–4.8, 0.70)	0.14	–1.7 (–4.6, 1.1)	0.24	–2.3 (–5.0, 0.4)	0.93
	Barriers to activity	–4.5 (–7.4, –1.5)	0.003	–4.6 (–8.4, –0.9)	0.02	–3.5 (–6.4, –0.7)	0.02
	Disinhibited eating	–2.0 (–7.0, 3.10)	0.45	–3.1 (–7.3, 1.2)	0.16	–3.6 (–8.3, 1)	0.12
Diabetes Knowledge Test	Knowledge	2.2 (–0.07, 4.5)	0.06	1.8 (–0.7, 4.2)	0.15	0.55 (–3.2, 4.3)	0.77

Intervention group *n* = 54, and control *n* = 60.

* Statistical analysis was performed using ancova, adjusting for imbalances at baseline between groups.

PCS, Physical Components of Health; MCS, Mental Components of Health.

## Discussion

In this educational intervention, we promoted principles of self-titration of insulin based on liberal diet and physical activity. No significant weight gain or deterioration in blood pressure, lipid profile, insulin doses or BMI was observed with this approach. Our study adds to the body of evidence demonstrating that promotion of dietary liberalization does not lead to a decline in HbA_1c_[3,6–8] or increase in serious hypoglycaemic episodes [26,27].

### Glycaemic control

HbA_1c_ improved significantly from baseline in both intervention and control groups at 3, 6 and 12 months. However, there were no statistically significant differences between the two groups, failing to replicate improvements in other studies [3,6–8]. There are a number of possible explanations for this.

First, all participants were already on multiple daily injections and many participants had received carbohydrate estimation and insulin titration training as part of their routine care. Although knowledge is a precondition for self-efficacy and not an indicator of it, the lack of improvement in knowledge score suggests self-efficacy skills may already have been common in this group of patients.

Second, the mean baseline HbA_1c_ was lower in this study than in the DAFNE and Düsseldorf studies. This suggests a reduced potential for lowering HbA_1c_ in this study, where no lower limit was set to ensure that the results were more widely applicable to standard diabetes care in the UK. However, lack of improvement in HbA_1c_ persisted even after excluding participants in intervention and control groups with a baseline HbA_1c_ < 7.5%.

Third, the delivery of our intervention in 2.5 days spread over 6 weeks may have diluted any positive impact on glycaemic control. The direct continuous support of other group members in a sustained intensive environment could well be the key to longer term behavioural change and glycaemic improvement achieved by other studies. It is also important to note that our intervention did not promote the use of a fixed algorithm to titrate insulin. Participants self-adjusted insulin doses according to their carbohydrate intake, level of activity, anticipation of exercise, climatic change on holiday, time zones and their insulin regime.

### Psychological outcomes

The BITES intervention led to some improvements in a number of psychological outcomes. Key areas of improvement were treatment satisfaction (DTS-Q) and self-empowerment (DES). Managing psychological aspects, setting and achieving goals and barriers to activity showed statistically significant improvements, suggesting success as a psychological intervention. The significant improvement in ‘barriers to activity’ score in the DHP scale is clinically relevant. This sub-scale addresses perceived barriers and operant anxiety resulting from diabetes, and the intervention positively influenced this. Although not statistically significant, ‘disinhibited eating’ and ‘psychological distress’ subscales of the DHP also improved.

Treatment satisfaction improved even in the absence of statistically significant glycaemic improvement. This could be due to poor correlation between these two variables, as suggested by previous studies [28]. However, it is important to note that HbA_1c_ did improve significantly in both arms of our study when compared with baseline, possibly influencing the perception of treatment satisfaction.

We used a large inventory of psychological instruments in assessing the psychological impact of the intervention. Discordant results noted in different scales (e.g. Treatment satisfaction vs. Fear of hypoglycaemia) in our study emphasize the need to design interventions with clear psychological objectives along with clinical objectives.

### Strengths and weaknesses

Our study has both strengths and weaknesses. We studied the impact of the educational intervention in a ‘real-world’ setting and did not specify cut-off thresholds for HbA_1c_. Although some details of the BITES curriculum are different from other brief interventions in the UK, the educational objectives [11] are similar.

We made every effort to ensure that the control group received less input than the intervention group. However, principles of self-management from the BITES course may have spilled over into the day-to-day practice of healthcare providers. No statistically significant intergroup differences were noted in frequency of use of general practitioner, hospital out-patients, specialist nurse or emergency service resources, nor was there any difference in acute or non-acute sick days.

Cluster randomization with multiple care delivery teams may overcome this, but ethical and practical considerations prevent such a study design in the UK. Moreover, open invitation may have led to recruitment of participants with high levels of motivation who may already have adopted many of the behaviours and principles that the course sought to instil. However, selection of patients based on their pre-intervention level of motivation would have reduced the clinical applicability of our study findings.

### Implications for clinical practice and research

There are up to 20 different interventions developed and used in different parts of the UK [11], with recommendations to offer such interventions to all patients with diabetes [2,29,30]. To improve patient access and optimize resource allocation, many of these interventions are delivered in 15–24 h over 4–6 weeks, compared with 35 h in five consecutive days as in the DAFNE and Düsseldorf studies. The lack of biophysical effectiveness seen in our study may warrant a closer examination of other brief interventions, although the content and delivery of these educational interventions are likely to differ.

Although delivered in a group setting, BITES provided considerable opportunity for individualized learning. Participants had to complete a workbook between sessions, and feedback was provided for workbook entries at the beginning of the subsequent session. As resource pressures often mandate provision of diabetes education in a group setting, further studies comparing the efficacy of one-to-one, group-based or hybrid learning methods have to be welcomed.

The interplay between biophysical markers of diabetes, psychological outcomes and perceptions of treatment satisfaction is complex [17,21–24], hence the use of a wide range of scales in our study to quantify psycho-educational outcomes. Although the inventories are often perceived to be practically challenging, all participants in our studies completed them and the feedback has been positive. We would therefore recommend inclusion of multiple relevant psychometric instruments in future studies in the domain of diabetes education.

## Conclusions

Our brief psycho-educational intervention promoting dietary liberalization in Type 1 diabetes showed no significant impact on HbA_1c_ or severe hypoglycaemia, but led to gains in treatment satisfaction and empowerment.

## Abbreviations

BMI: body mass index
CI: confidence interval
DAFNE: Dose Adjustment for Normal Eating
DES: Diabetes Empowerment Scale
DHP: Diabetes Health Profile
DKT: Diabetes Knowledge Test
DTS-Q: Diabetes Treatment Satisfaction Questionnaire
HbA_1c_: haemoglobin A_1c_
HFS: Hypoglycaemia Fear Scale
IPQ: Illness Perception Questionnaire
SF-36: 36-Item Short-form Health Survey
